# Establishment of a regional Mpox surveillance network in Central Africa: shared experiences in an endemic region

**DOI:** 10.1186/s41256-025-00408-y

**Published:** 2025-03-05

**Authors:** Emmanuel Hasivirwe Vakaniaki, Sydney Merritt, Sylvie Linsuke, Emile Malembi, Francisca Muyembe, Lygie Lunyanga, Andrea Mayuma, Papy Kwete, Thierry Kalonji, Joule Madinga, Matthew LeBreton, Emmanuel Nakoune, Ernest Kalthan, Sevidzem Shang, Julius Nwobegahay, Odianosen Ehiakhamen, Elsa Dibongue, Jean-Médard Kankou, Bernard Erima, Denis K. Byarugaba, Paige Rudin Kinzie, Franck Mebwa, Francis Baelongandi, Aimé Kayolo, Pépin Nabugobe, Dieudonné Mwamba, Jean Malekani, Beatrice Nguete, Didine Kaba, Lisa E. Hensley, Jason Kindrachuk, Laurens Liesenborghs, Robert Shongo, Jean-Jacques Muyembe-Tamfum, Nicole A. Hoff, Anne W. Rimoin, Placide Mbala-Kingebeni

**Affiliations:** 1 Department of Epidemiology and Global Health, Institut National de Recherche Biomédicale (INRB), School of Medicine, University of Kinshasa (UNIKIN), Kinshasa, Democratic Republic of the Congo; 2 Department of Clinical Sciences, Institute of Tropical Medicine, Antwerp, Belgium; 3 Department of Epidemiology, Jonathan and Karin Fielding School of Public Health, University of California, Gordon-Levin Endowed Chair in Infectious Diseases and Public Health, UCLA Fielding School of Public Health, 650 Charles E. Young Dr, CHS 41-275, Los Angeles, CA 90095 USA; 4 National Program for Monkeypox and Viral Hemorrhagic Fevers, Ministry of Health, Kinshasa, Democratic Republic of the Congo; 5 Mosaic, Yaoundé, Cameroon; 6 Pasteur Institute of Bangui, Bangui, Central African Republic; 7 Ministry of Health and Populations, Bangui, Central African Republic; 8 Military Health Research Center (CRESAR), Yaoundé, Cameroon; 9 Nigeria Centre for Disease Control and Prevention, Abuja, Nigeria; 10 Cameroon National Program for the Prevention and Control of Emerging and Re-Emerging Zoonoses, Ministry of Health, Yaoundé, Cameroon; 11 Epidemiology and Disease Control Department, Ministry of Public Health, Brazzaville, Republic of Congo; 12 Makerere University, Kampala, Uganda; 13 Purdue University, West Lafayette, IN USA; 14 Division Provinciale de La Santé de La Tshopo, Kinshasa, Democratic Republic of the Congo; 15 Epidemiological Surveillance Department, Kinshasa, Ministry of Health, Kinshasa, Democratic Republic of the Congo; 16 National Institute of Public Health, Kinshasa, Ministry of Health, Kinshasa, Democratic Republic of the Congo; 17 Department of Biology, Faculty of Science, University of Kinshasa, Kinshasa, Democratic Republic of the Congo; 18 Kinshasa School of Public Health, University of Kinshasa, Kinshasa, Democratic Republic of the Congo; 19 Zoonotic and Emerging Disease Research Unit, National Bio and Agro-Defense Facility, USDA Agricultural Research Service (ARS), Manhattan, KS USA; 20 Department of Medical Microbiology & Infectious Diseases, Max Rady College of Medicine, University of Manitoba, Winnipeg, Canada; 21 Department of Microbiology, Immunology and Transplantation, KU Leuven, Leuven, Belgium; 22 Microbiology Service, Department of Medical Biology, Cliniques Universitaires de Kinshasa, University of Kinshasa (UNIKIN), Kinshasa, Democratic Republic of the Congo

**Keywords:** Mpox, Monkeypox, Regional surveillance network, Shared experience, The Democratic Republic of Congo

## Abstract

To address the underreporting of mpox cases in endemic regions, a regional surveillance network, known as the Mpox Threat Reduction Network (MPX-TRN), was established between five neighboring countries in Central and West Africa in 2022. One direct outcome of the MPX-TRN has been the strengthening of regional mpox surveillance. This consortium has facilited open communication channels, detection of cross-border mpox cases, and improvements of the detection and diagnosis of mpox in Central Africa and worldwide. Importantly, the MPX-TRN provides a scalable model for addressing underreporting of diseases, such as mpox.

**Supplementary Information:**

The online version contains supplementary material available at 10.1186/s41256-025-00408-y.

## Background

Although mpox outbreaks have occurred outside of Africa–including the unprecedented worldwide epidemic in 2022–the disease has long been considered endemic in Central and West Africa [1, 2]. Prior to this epidemic, the DRC reported more than 80% of global cases, followed by Nigeria, which has experienced a resurgence in cases since 2017, after 39 years without a reported case [1]. Until recently, there has been limited interest and minimal funding for mpox research and response from the international health community [3]. In endemic regions, isolated mpox surveillance systems and varying treatment frameworks, with limited regional standardization had further complicated accurate reporting. A lack of cross-border communication channels is yet another challenge to understanding and tracking mpox cases. [3]. While mpox surveillance among high-income regions was enacted quickly through public health entities in 2022, most surveillance within endemic areas relies on research-oriented investigations supported by piecemeal funding [4].

Despite the rapid increases in cases, and geographic expansion in non-endemic regions of the 2022 global outbreak, no parallel increase in cases was observed among endemic countries. International reporting guidelines were updated to include only laboratory-confirmed cases; yet, endemic regions had typically only reported suspected cases due to resource limitations. For example, as the epicenter of the disease in Africa, the DRC reported annual suspected cases totaling 6216, 2841, and 5697 from 2020 to 2022. Laboratory confirmation from samples was undertaken for less than 10% of these cases [5, 6]. Thus, these changes in global reporting guidelines have likely led to significant under-reporting of the actual burden of mpox within resource limited settings. Under-reporting of mpox cases can have serious public health implications–such as the allocation of surveillance resources, delays in outbreak response, and ultimately, potential misdirection of vaccination deployment strategies.

In 2023, the DRC reported over 14,000 suspected cases yet fewer than 2,000 samples were sent for laboratory confirmation due to ongoing resource limitations [6]. In the same period, the Central African Republic (CAR) notified 130 suspected cases with all 130 tested and 19 confirmed; Cameroon reported 114 suspected cases, 27 were confirmed; and Nigeria tested over 4,000 samples with a 27% positivity rate [7–9]. As of week 14, 2024, the Republic of the Congo (ROC) notified 59 suspected cases; 32 were tested, and 19 confirmed. Varying laboratory access and confirmation rates can yet again be attributed to historic research-driven funding and investigations as opposed to widespread surveillance practices. Despite increased global awareness of mpox, funding in resource-limited areas has remained stagnant.

## Mpox regional network

To enhance mpox detection capacity and address under-reporting, the Mpox Threat Reduction Network (MPX-TRN) was launched in January 2022. This network is part of an ongoing partnership between the University of California, Los Angeles (UCLA), the DRC National Institute of Biomedical Research (INRB), and Mosaic (Cameroon) with broader objectives including: improving sequencing for detecting cross-border transmission of mpox, collecting samples with differential pox virus exposures, conducting environmental sampling for mpox, supporting surveillance in sites for improved mpox case confirmation in collaboration with the National Program for the control of Mpox and Viral Hemorrhagic Fevers (PNLMPX-VHF), and creating a regional network to enhance capacity for a sustainable biosurveillance system.

**Fig. 1:**
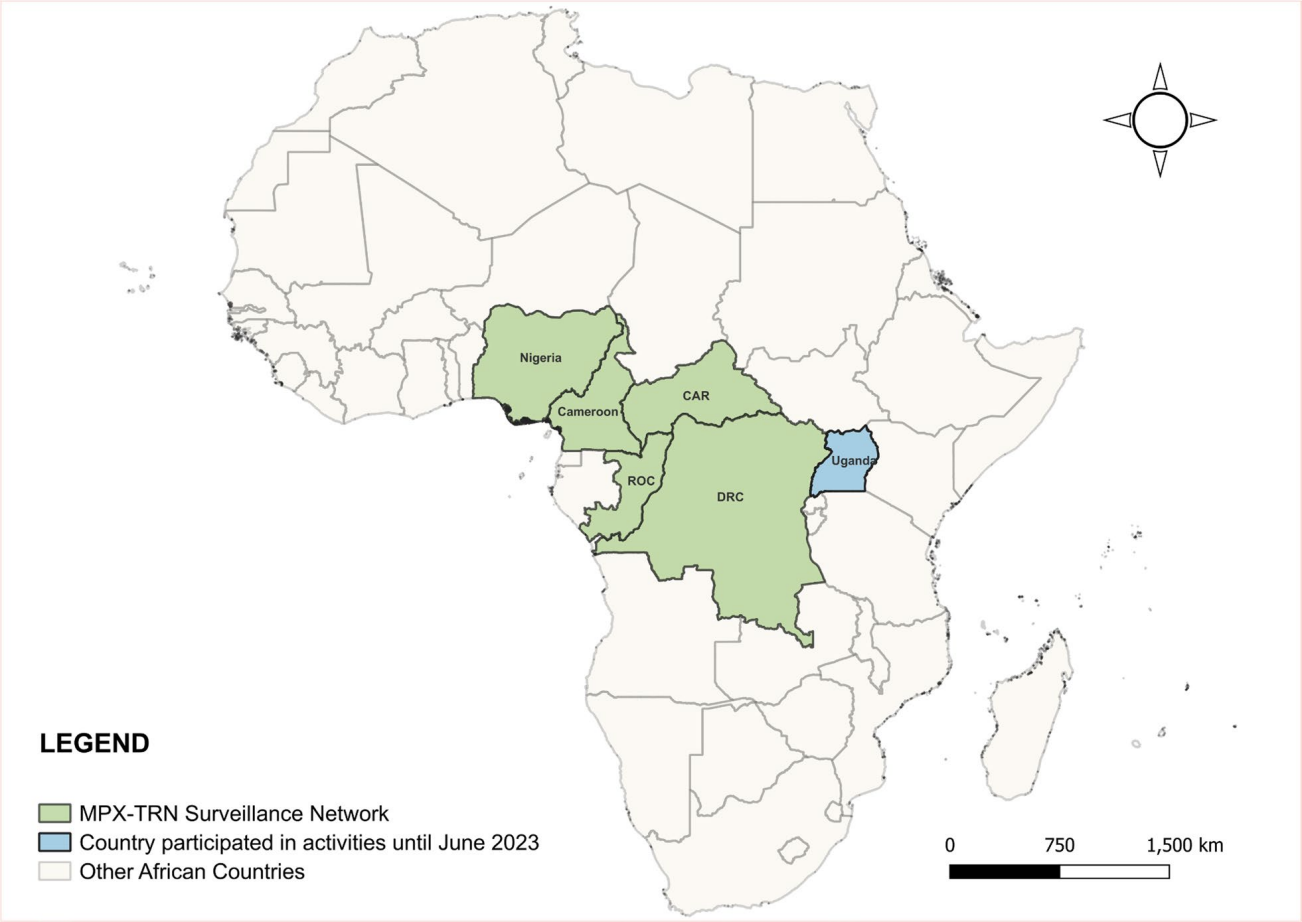
Map of mpox regional surveillance network countries

Ultimately, the final objective of the MPX-TRN is to support and strengthen regional collaboration and training in mpox surveillance. Leveraging the extensive experience of the INRB, in collaboration with the PNLMPX-VHF, the DRC team organized two events that included regional experts and partners: Mosaic, PNLMPX-VHF, the Kinshasa School of Public Health (KSPH), and the Centers for Disease Control and Prevention (CDC); the Disease Control Directorate and the Pasteur Institute of Bangui, CAR; the National Program for the Prevention and Control of Emerging and Re-emerging Zoonotic Diseases and the Military Health Research Center Cameroon; the Directorate of Disease Control and the Public Health Laboratory, ROC; the Nigeria Center for Disease Control and Prevention; and initially, Makerere University, Uganda (Fig. 1).

In August 2022, participants from seven countries, including, DRC, ROC, CAR, Cameroon, Nigeria, Uganda, and the USA assembled for a two-day meeting. Participants from each country could discuss their mpox surveillance system and key components to be included in creating a regional network (Additional file 1: Fig. S1). In November 2022, a larger two-week meeting expanded on this training, with sessions both in Kinshasa at the INRB as well as Kisangani, Tshopo province – the DRC’s first mpox sentinel surveillance site.

While in Kinshasa, the group discussed their format for case investigations, case definitions, laboratory testing, sequencing procedures and an overview of bioinformatic analysis. Operationally, the MPX-TRN framework supported regional training of best practices in mpox surveillance and control. In Kisangani, the team participated in ongoing mpox investigations, learned how surveillance and patient care is conducted in DRC health facilities, and observed how samples are collected and shipped to INRB in Kinshasa (Additional file 2: Fig. S2 A & B). The team also visited the site’s laboratory where work is ongoing to validate an mpox/orthopox GeneXpert test.

## Outcomes

Since the inception of the regional network for the MPX-TRN, three primary outcomes have emerged. First, the MPX-TRN facilitated the first regular communication focused solely on mpox surveillance in the Central and West African countries and their partners. Second, it fostered open collaboration for improved detection and communication of cross-border mpox transmission. This partnership was particularly effective in 2023 when multiple outbreaks circulating between the DRC, CAR, and ROC were identified on both sides. Specifically, clusters in Mbaya health zone, DRC were epidemiologically linked with past CAR mpox outbreaks in January 2023 (10). Finally, the network has served as the foundation to enable larger, more formalized forms of collaboration, such as joint funding opportunities for enhanced disease detection and research-focused grants. The regional surveillance network established and maintained WhatsApp channels to maintain real-time communication and share updates on publications and reports. These channels were not designed to serve as a replacement for the official channels, but to share real-time open-source information for rapid action. Beyond these channels, the network implemented additional bi-annual virtual meetings where participants could provide updates on trends, changes in surveillance, and ongoing research studies.

Ultimately, the MPX-TRN provided a set of responsibilities for all members–continued open communication, participation in bi-annual mpox data sharing meetings, and collaborative cross-border case tracking. Establishing the MPX-TRN has faced some challenges. Namely, funding for the MPX-TRN cannot support expansive regional mpox surveillance. Scheduling wasn’t without inconvenience–coordinating formal meeting times has been a challenge. This was best circumvented by relying on the WhatsApp channels.

## Conclusions

Establishing regional surveillance network through the MPX-TRN has created a robust framework for regional collaboration on locally-driven mpox research. Such consortia can continue to promote open communication and experiences, supporting the political vision of African governments to reduce the impact of mpox in Central and West Africa. Beyond initial establishment, the MPX-TRN can be expanded to include other countries in the region such as Burundi and Kenya as more mpox cases are reported. Uniquely, the activities of the MPX-TRN are relatively low-cost, and instead more a formal connection of regional partners. Notwithstanding, longevity and sustained communication could be extended through formal joint research activities at these border regions. This type of regional support group could serve as a model for other regions with porous borders and underreporting of other neglected endemic diseases.

## Supplementary Information

**Additional file 1. Figure 1.** Kick-off meeting of MPX-TRN by Deputy Minister of the DRC's Ministry of Public Health, Hygiene and Prevention at INRB, August 2022.

**Additional file 2. Figure 2.** (A) Regional workshop on MPX surveillance, Yakusu General Referral Hospital, Tshopo province, DRC, November 2022. (B) Laboratory diagnostics using RT-PCR for mpox identification at INRB, Kinshasa, November 2022.

## Data Availability

All data and information presented in this perspective is openly available as part of regular mpox surveillance activities in DRC and neighboring countries.

## Declarations

### Reflexivity Statement

This manuscript, and the corresponding establishment of the MPX-TRN, was a highly collaborative initiative including international leaders in mpox surveillance (including but not limited to leaders of national laboratories in the Democratic Republic of the Congo, Central African Republic, Cameroon, Nigeria, and the Republic of Congo) as well as those directly involved with mpox case investigations and sampling, such as the DRC’s National Program for Mpox and Viral Hemorrhagic Fever team members. The breadth of the author affiliations is reflective of the inclusive and collaborative nature of this project. Since it’s inception, the MPX-TRN has contributed not only to increased local capacity through cross-border training, but also at a higher level, improved mpox-related health system communications.
