# Supplementary figures and images for: Establishment of a regional Mpox surveillance network in Central Africa: shared experiences in an endemic region
Source: Glob Health Res Policy. 2025 Mar 5;10:14. doi: 10.1186/s41256-025-00408-y (PMC11881381; doi:10.1186/s41256-025-00408-y)

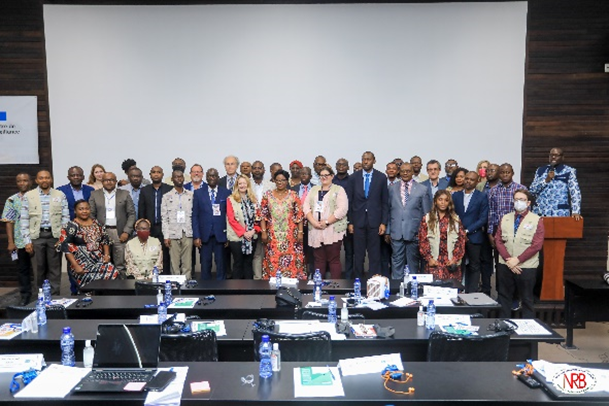

Supplement: Supplementary file 1 — Additional file 1. Figure 1. Kick-off meeting of MPX-TRN by Deputy Minister of the DRC's Ministry of Public Health, Hygiene and Prevention at INRB, August 2022. [file 41256_2025_408_MOESM1_ESM.docx]

**A**


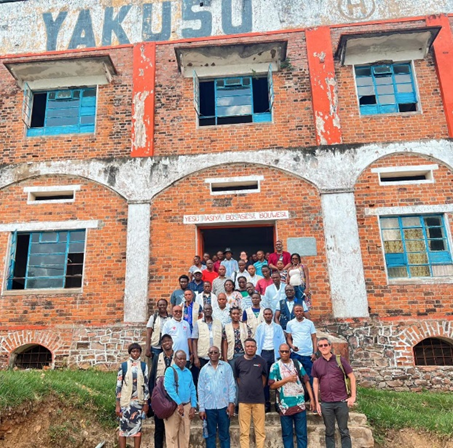


**
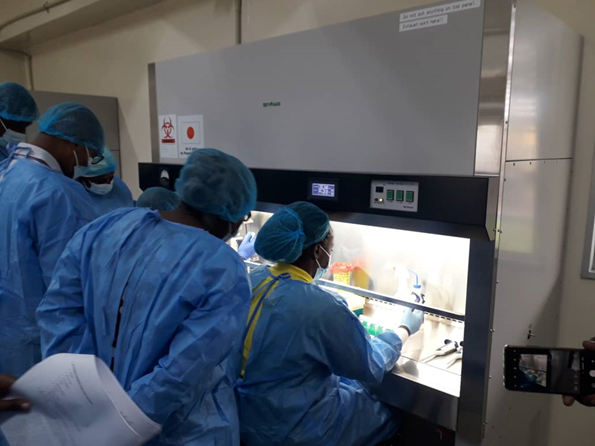
B**

Supplement: Supplementary file 2 — Additional file 2. Figure 2. (A) Regional workshop on MPX surveillance, Yakusu General Referral Hospital, Tshopo province, DRC, November 2022. (B) Laboratory diagnostics using RT-PCR for mpox identification at INRB, Kinshasa, November 2022. [file 41256_2025_408_MOESM2_ESM.docx]
